# circRNA_0000140 suppresses oral squamous cell carcinoma growth and metastasis by targeting miR-31 to inhibit Hippo signaling pathway

**DOI:** 10.1038/s41419-020-2273-y

**Published:** 2020-02-10

**Authors:** Qiu-Shi Peng, Ya-Nan Cheng, Wen-Bai Zhang, Hui Fan, Qiu-Hua Mao, Pu Xu

**Affiliations:** Stomatological Center of Hainan Province, Affiliated Haikou Hospital, Xiangya School of Medicine, Central South University, Haikou, 570208 China

**Keywords:** Oral cancer, Cancer

## Abstract

Oral squamous cell carcinoma (OSCC) is one of the most common malignancies and has a poor prognosis. Circular RNA (circRNA) has been increasingly recognized as a crucial contributor to carcinogenesis. circRNA_0000140 has been aberrantly expressed in OSCC, but its role in tumor growth and metastasis remains largely unclear. Sanger sequencing, actinomycin D, and RNase R treatments were used to confirm head-to-tail junction sequences and the stability of circ_0000140. In vitro cell activities, including proliferation, migration, invasion, and apoptosis, were determined by colony formation, transwell, and flow cytometry assays. The expression levels of circ_0000140, Hippo signaling pathway, and serial epithelial–mesenchymal transition (EMT) markers were measured by quantitative real-time PCR, western blotting, immunofluorescence, and immunohistochemistry. Dual luciferase reporter assays and Argonaute 2-RNA immunoprecipitation assays were performed to explore the interplay among circ_0000140, miR-31, and LATS2. Subcutaneous tumor growth was observed in nude mice, in which in vivo metastasis was observed following tail vein injection of OSCC cells. circ_0000140 is derived from exons 7 to 10 of the *KIAA0907* gene. It was down-regulated in OSCC tissues and cell lines, and correlated negatively with poor prognostic outcomes in OSCC patients. Gain-of-function experiments demonstrated that circ_0000140 enhancement suppressed cell proliferation, migration, and invasion, and facilitated cell apoptosis in vitro. In xenograft mouse models, overexpression of circ_0000140 was able to repress tumor growth and lung metastasis. Furthermore, mechanistic studies showed that circ_0000140 could bind with miR-31 and up-regulate its target gene *LATS2*, thus affecting OSCC cellular EMT. Our findings demonstrated the roles of circ_0000140 in OSCC tumorigenesis as well as in metastasis, and circ_0000140 exerts its tumor-suppressing effect through miR-31/LATS2 axis of Hippo signaling pathway in OSCC.

## Introduction

Oral squamous cell carcinoma (OSCC) accounts for more than 95% of all head and neck cancer and ranks among the top eight causes of cancer-related death globally^1,2^. The deadliest aspect of oral cancer is metastasis^3,4^. Once the tumor is formed, cells may begin to break off from this tumor and migrate to other parts of the body^5^. These cancer cells that travel through the body established new tumors in remote locations like lung metastasis^6,7^. Less than 50% of patients will survive for 5 years, and majority of patients with metastatic OSCC die within 1 year^5,8^. Understanding the molecular mechanisms underpinning OSCC metastasis is of crucial importance to the development of specific diagnostic methods and individualized therapeutic strategies.

Hippo pathway is a conserved pathway that controls cell proliferation, organ size, tissue regeneration, and stem cell self-renewal by regulating the downstream transcriptional coactivators YAP and TAZ^9–11^. Among the core kinase components of the Hippo pathway, the Dbf2-related kinases LATS1 and LATS2 (LATS1/2) have gained increasing attention. LATS1/2 have emerged as key regulators of various oncogenic or tumor-suppressive effectors, as well as epithelial–mesenchymal transition (EMT) mediators during cancer metastasis^9^. For instance, LATS2 was reported to increase cell invasive capacities of metastasis via harboring mutant p53, leading to increased SNAIL1 stability in breast cancer^12^. In OSCC, lower LATS2 expression was observed in OSCC patients^13^, and overexpression of LATS2 blocked cell proliferation, colony formation, and cell invasion in vitro, and xenografts in vivo^14^. Therefore, understanding the molecular mechanism of LATS2-mediated Hippo pathway in OSCC will provide significant information for the treatment of OSCC.

Circular RNAs (circRNAs) are a class of RNA that is widespread and abundant and generally formed by alternative splicing of pre-messenger RNA (mRNA)^15^. Recent studies showed great potential of circRNAs as promising clinical biomarkers and therapeutic targets for cancers. Moreover, the involvement of the circRNA-miRNA-mRNA signaling axis in OSCC development and progression has been established^16^. Of note, hsa_circ_0008309 inhibits miR-136-5p and miR-382-5p expression and augments ATXN1 expression in OSCC^17^. circRNA_100290, on the other hand, functions as a competing endogenous RNA (ceRNA), which regulates other RNA transcripts by competing for shared microRNAs (miRNAs), to antagonize miR-378a-driven GLUT1 suppression, finally leading to the induction of glycolysis and cell proliferation in OSCC^18^. While significant lower expression of circ_0000140 was found in OSCC patients^19^, the molecular mechanism behind this is still unclear.

miRNAs are members of small non-coding RNA molecules of ~22 nucleotides long that play essential regulatory functions in cancer development^20^. Increasing evidence supported that miR-31 tightly associated with tumorigenesis and metastasis, with miR-31 functioning either as an oncogene or as a tumor suppressor in a context-dependent manner^21,22^. In the context of OSCC, miR-31 is highly expressed and its tumor-promoting function in OSCC cells has been correlated with SIRT3 or ACOX1^23,24^. Meanwhile, miR-31 has been shown to cause reduction of LATS2 expression and protein levels and activating EMT in cell lines derived from lung cancer^25^. It is likely that similar molecular mechanism exists for OSCC development.

Lung metastasis from the oral cavity mainly occurs in the advanced stages and careful examination of the patient during primary cancer treatment has a significant impact on a patient’s life^4^. However, few studies of circRNAs and miRNAs have been reported to be involved in both OSCC tumorigenesis and metastasis. In the present study, we proposed a molecular mechanism for circ_0000140 and miR-31 in the lung metastasis of OSCC and our results suggested that circRNAs and miRNAs are potential targets for the treatment of OSCC.

## Materials and methods

### Clinical tissues

Fifty-six paired OSCC and adjacent normal tissues, together with lymph node metastasis samples, were obtained from patients who received surgery in Haikou Hospital Affiliated to Xiangya Medical College of Central South University. None of them received chemoradiotherapy prior to surgery. The study protocol was approved by the Medical Ethics Committee of Haikou Hospital Affiliated to Xiangya Medical College of Central South University, with written informed consent obtained from all patients. The relevant clinical information was provided in Table 1.

**Table 1:** Correlation between circ_0000140 expression and clinicopathological characteristics of OSCC patients.

Characteristics	Group	Cases (*n*)	circ_0000140 expression		*P* value
			Low (*n* = 28)	High (*n* = 28)	
Gender	Female	22	10	12	0.781
	Male	34	18	16	
Age	≤60	20	8	12	0.403
	>60	36	20	16	
Smoking	Non-smoker	25	11	14	0.591
	Smoker	31	17	14	
Tumor site	Tongue	16	10	6	0.375
	Non-tongue	40	18	22	
Drinking	Non-drinker	26	15	11	0.422
	Drinker	30	13	17	
Lymph node metastasis	No	26	8	18	0.015*
	Yes	30	20	10	
TNM stage	I–II	27	9	18	0.031*
	III–IV	29	19	10	
Tumor grade	Well/moderate	37	15	21	0.163
	Poor	19	13	7	

*P < 0.05.

### Cell culture

Human oral keratinocyte (HOK) cells and four human OSCC cell lines (Cal-27, SCC-25, SCC-9, and HSC-3) used in this study were purchased from American Type Culture Collection (Manassas, VA, USA). The HOK cells were cultured in oral keratinocyte growth medium (ScienCell, Carlsbad, CA, USA), Cal-27 and SCC-25 cells were cultured in Dulbecco’s modified Eagle’s medium (DMEM), while HSC-3 and SCC-9 cells were cultured in DMEM/F12 medium. Cells were tested without contamination with mycoplasma. All mediums mentioned above were supplemented with 10% fetal bovine serum (FBS; Hyclone, Israel) and 100 U/ml penicillin and streptomycin (Invitrogen, Camarillo, CA, USA). Cells were maintained at 37 °C in an atmosphere filled with 5% CO_2_.

### Fluorescence in situ hybridization

HOK cells (1 × 10^5^) were fixed in 4% paraformaldehyde (PFA) for 10 min and subsequently washed with phosphate-buffered saline (PBS). Cells were then permeabilized with 0.5% Triton X-100 in precooled PBS for 15 min at 4 °C. Digoxigenin (DIG)-labeled circ_0000140 probe or control probe mixture was performed to incubate cells for 4 h at 37 °C. After briefly washing with 2× saline sodium citrate for 5 min in the dark, the signal was detected using horseradish peroxidase (HRP)-conjugated anti-DIG secondary antibodies (Jackson, West Grove, PA, USA). Olympus confocal laser scanning microscope was used to obtain the image. 4′,6-Diamidino-2-phenylindole (DAPI) was used for nuclear counterstaining.

### Sanger sequencing, actinomycin D, and RNase R treatment

The circ_0000140 sequence was obtained using divergent primers sent to Sangon (Shanghai, China) for Sanger sequencing analysis. To block transcription, 2 mg/ml actinomycin D or dimethylsulfoxide (Sigma-Aldrich, St. Louis, MO, USA) as a negative control was added into the cell culture medium. For RNase R treatment, total RNA (2 μg) was incubated for 1 h at 37 °C with or without 3 U/μg of RNase R (Epicentre Technologies, Madison, WI, USA). After treatment with actinomycin D and RNase R, quantitative real-time PCR (qRT-PCR) was performed to determine the expression levels of KIAA0907 and circ_0000140 mRNA.

### Total RNA extraction and real-time PCR

Total RNA was isolated from OSCC tissues and cell lines with the use of TRIzol reagent (Invitrogen, Carlsbad, CA, USA). Complementary DNA (cDNA) was synthesized using random primers and the SuperScriptIII reverse transcriptase (Quantabio, Beverly, MA USA). qRT-PCR analysis was performed using Bulge-Loop miRNA qRT-PCR Starter Kit (RiboBio), with U6 serving as an internal normalized reference. For detection of circ_0000140 and mRNAs, reverse transcription reactions were carried out using Prime Script RT Reagent Kit (Takara Bio, Shiga, Japan), with glyceraldehyde 3-phosphate dehydrogenase (GAPDH) used as an internal control. The PCR reaction was run in triplicate with 7500 Real-Time PCR System (Applied Biosystems, Foster City, CA, USA) using SYBR Premix Ex Taq II (TaKaRa). The amplification comprised of a 5 min denaturation at 95 °C, followed by 40 cycles of denaturation at 95 °C for 15 s, annealing at 55 °C for 30 s, and extension at 60 °C for 1 min. The primer sequences used were as follows: 5′-AGACTCAAGACGAGGTGAGGAG-3′ (forward) and reverse 5′-ATGACCCAAACCCACTAATAAA-3′ (reverse) for KIAA0907 expression; 5′-CCGGCATTACCTACTGGAGTC-3′ (forward) and 5′-CCTTCCACCTTCTCCTTGACA-3′ (reverse) for circ_0000140 expression; 5′-ACACTCCAGCTGGGTAGCAGCGGGAACAGTTC-3′ (forward) and 5′-CTCAACTGGTGTCGTGGA-3′ (reverse) for miR-31 expression; 5′-CAGCAGCTGCCAGACCTATT-3′ (forward) and 5′-AGGATATGGAGGTGGTGGCT-3′ (reverse) for LATS1 expression; 5′-AGATTTCGGCCTCTGCACTG-3′ (forward) and 5′-TAGGGTCTTCAGCCTGTCCC-3′ (reverse) for LATS2 expression; 5′-CTCGCTTCGGCAGCACA-3′ (forward) and 5′-AACGCTTCACGAATTTGCGT-3′ (reverse) for U6 expression; and 5′-CCAGGTGGTCTCCTCTGA-3′ (forward) and 5′-GCTGTAGCCAAATCGTTGT-3′ (reverse) for GAPDH expression. The relative expression levels were calculated using the 2−^∆∆Ct^ method after normalization with reference control.

### Cell transfection

The full-length circ_0000140 was cloned into overexpression plasmids. miR-31 mimics (sense: 5′-AGGCAAGAUGCUGGCAUAGCU-3′; antisense: 3′-CUAUGCCAGCAUCUUGCCUUU-5′), miR-31 inhibitors (5′-AGCUAUGCCAGCAUCUUGCCU-3′), and plasmids were purchased from Genema (Shanghai, China). Short hairpin RNA (shRNA) against LATS2 (shLATS2: 5′-GGACCTCACTGCATTAAA-3′) and negative control shRNA (sh-NC: 5′-CGTACGCGGAATACTTCGA-3′) were synthesized by Genema (Shanghai, China). circ_0000140 gene and LATS2 3′-untranslated region (UTR) fragment containing putative binding sites for miR-31 reporter vector were synthesized by Genechem (Shanghai, China). The cells (5 × 10^5^) were plated in 6-well plates 24 h prior to transfection with circ_0000140 overexpression plasmid, miR-31 mimics, miR-31 inhibitors, and shRNAs with 60–70% confluence, and then transfected using Lipofectamine 2000 (Invitrogen, Carlsbad, CA, USA). For transient transfection assay with shRNAs and plasmids, cells were harvested at 48 h for further experiments. Stable cell clones were chosen by appropriate antibiotics (puromycin, 2–5 μg/ml, Sigma) for at least 1 week after virus infection or plasmid transfection.

### RNA immunoprecipitation

RNA immunoprecipitation (RIP) assay was conducted using the EZMagna RIP Kit (Millipore, Billerica, MA, USA) according to the manufacturer’s instruction. The Argonaute 2 (AGO2)-RIP experiments were carried out in Cal-27 and HSC-3 cells transiently expressing circ_0000140 and miR-31. RIP lysis buffer with proteinase and RNase inhibitors were used to lyse the OSCC cells. Magnetic beads conjugated with human anti-Ago2 antibody or control anti-immunoglobulin G (IgG) antibody were performed by incubating the RIP lysates. The lysates were then harvested at 4 °C for 6 h. The purified RNA was subjected to qRT-PCR and gel-staining analyses as proteins were removed from the beads.

### Immunofluorescence

After treatment as indicated, cells were fixed with 4% PFA, permeabilized with 0.01% Triton X-100, and blocked in 2% bovine serum albumin (BSA), followed by overnight incubation with YAP1 primary antibodies. Secondary fluorescent antibodies were added and incubated for a further 1 h. DAPI was used for nuclear counterstaining. Images were captured by an SP5 confocal microscope (Leica Microsystems, Buffalo Grove, IL, USA).

### Colony formation assay

For the colony formation assay, after treatment with mimics, inhibitors, or vectors, the cells were trypsinized and 1000 viable cells were plated into 6-well plates. After 2 weeks in culture, the media were removed, and the cells were fixed in 96% ethanol for 10 min. Cells were then stained with 0.5% crystal violet for 30 min and scored using a microscope and the ImageJ software (1.47V, NIH, USA).

### Annexin V/PI assay

Cal-27 and HSC-3 cells transfected with circ_0000140 overexpression plasmid, miR-31 mimics, miR-31 inhibitors, or shLATS2 were cultured for 48 h. The cells were washed twice in ice-cold PBS and resuspended in 500 µl 1× Annexin binding buffer to a concentration of 1 × 10^6^/ml. The cells were then incubated with 5 µl 1× Annexin V/FITC and 10 µl propidium iodide (PI) for 15 min at room temperature in dark. Flow cytometry was performed with a BD LSRII instrument (BD Biosciences, Bedford, MD, USA). Appropriate quadrant was assigned, and the percentages of early and late apoptosis indicated by Annexin V and PI staining were calculated.

### Cell migration and invasion assay

For cell migration assay, transfected cells harvested in serum-free medium were placed into the upper chamber of Boyden Transwell chambers (Corning, Cambridge, MA, USA) with an 8-μm pore membrane in a 24-well format. For cell invasion assay, cells were seeded in the upper chambers pre-coated with 100% Matrigel (BD Biosciences). Complete medium containing 10% FBS in the lower chambers served as a chemoattractant. The cells remaining on the upper surface were gently removed with a cotton swab and those located on the lower chamber were fixed, stained with crystal violet, and counted in at least five random fields under a light microscope (Olympus Corporation, Tokyo, Japan).

### Western blot analysis

Total proteins were extracted from tissues and cells by RIPA buffer and the concentration was determined by the BCA Protein Assay Kit. Protein (30 μg) was separated by 10% sodium dodecyl sulfate-polyacrylamide gel electrophoresis and then transferred to polyvinylidene difluoride membranes. The membranes were blocked with 1% BSA in TBS buffer and primary antibodies were incubated at 4 °C. The membrane was washed with 1× TBST and incubated with a secondary antibody conjugate in 1× TBS for 1 h at room temperature. The membrane was washed with 1× TBST and the relative levels of each protein were quantified with the Quantity One software (Bio-Rad, California, USA). GAPDH detected on the same blot served as a loading control. The primary antibodies were obtained from Cell Signaling Technology as follows: GAPDH (D16H11), MMP-2 (D4M2N), MMP-9 (D6O3H), vimentin (D21H3), E-cadherin (24E10), N-cadherin (D4R1H), LATS2 (D83D6), p53 (DO-7), TAZ (E8E9G), p-TAZ (E1X9C), YAP1 (#4912), and p-YAP1 (D9W2I).

### Dual luciferase reporter assay

Serial constructs containing circ_0000140 or LATS2 3′-UTR were generated using the pGL3 luciferase promoter plasmid (Promega Corporation, Madison, WI, USA). Point mutations of the miR-31-targeting sites in the circ_0000140 or LATS2 3′-UTR were directly synthesized using the QuickChange Multiple Site-directed Mutagenesis Kit (Stratagene, La Jolla, CA, USA). Each plasmid construct was subsequently co-transfected with miR-31 mimics, miR-31 inhibitors, and a corresponding negative control into Cal-27 and HSC-3 cells seeded in 12-well plates by using the Lipofectamine 2000 method (Invitrogen, Carlsbad, CA, USA). After transfection for 48 h, cells were harvested, and luciferase activities were detected by the Dual Luciferase Reporter Assay Kit (Promega). The ratio of firefly to Renilla luciferase activity was subsequently determined.

### Tumor xenografts and tail vein injection experiments

All animal experiments were performed in accordance with the National Institutes of Health Guide for the Care and Use of Laboratory Animals and approved by the Institutional Animal Care and Use Committee of Haikou Hospital Affiliated to Xiangya Medical College of Central South University. Six-week-old female BALB/c nude (nu/nu) mice were purchased from SJA Laboratory Animal Co., Ltd (Hunan, China; *n* = 32) and maintained in a specific pathologic-free environment. For in vivo tumorigenic assay, BALB/c nude mice were randomly divided into four groups with eight mice in each group, and stable Cal-27 and HSC-3 cells overexpressing circ_0000140 or negative control were subcutaneously injected into the mice. The investigator was blinded to the group allocation during the experiment. The tumor diameters were measured by calipers every 5 days when tumor masses were identified. Tumor volume (*V*) is calculated as follows: *V* = 0.5 × *L* × *W*^2^, where *L* and *W* are defined as the tumor length (*L*) and width (*W*). At 30 days post injection, mice were euthanized, and the tumors were excised, photographed, and tissue sections were obtained for further immunohistochemical staining. For the tail vein injection experiment, stable Cal-27 and HSC-3 cells overexpressing circ_0000140 or negative control were injected into the tail veins of six mice. After 30 days of injection, the mice were sacrificed and the lungs were excised, photographed, and visible tumor nodes on the lung surface were counted.

### Immunohistochemistry

Briefly, after antigen retrieval, dewaxed and rehydrated tumor slides were treated with 3% hydrogen peroxide and then 5% BSA. The slides were then incubated overnight with primary antibody anti-LATS2 (Cell Signaling Technology, USA), anti-Ki-67 (Abcam, USA), anti-MMP-9 (Abcam, USA), and anti-YAP1 (Abcam, USA), and subsequently incubated with MaxVisionTM HRP-Polymer IgG complexes (KIT5150; Maixin-Bio, Shenzhen, China). A secondary antibody was then applied for 30 min at room temperature, and the sections were counterstained with hematoxylin and observed under a microscope.

### Hematoxylin and eosin staining

The lungs tissues were fixed intra-tracheally with 4% PFA in phosphate buffer, and embedded in paraffin. Sections (4 μm) were stained with hematoxylin and eosin, and then examined with a Philips CM10 (Philips, Zurich, Switzerland) 400 electron microscope.

### Statistical analysis

All experiments were performed at least three times, and all values are presented as the mean ± standard deviation (SD). Statistical analysis was performed by GraphPad Prism 6 (GraphPad Software Inc.). All data were in a normal distribution, and variance was similar between the groups that are being statistically compared. Unpaired two-tailed Student’s *t* test was used to compare the difference between two groups. One-way analysis of variance followed by Tukey’s post hoc test was used for multiple comparisons. The correlation between circ_0000140 expression and clinicopathological characteristics of OSCC patients was assessed by the *χ*^2^ test. Spearman’s correlation analysis was performed to analyze the correlation among circ_0000140, miR-31, and LATS2 in OSCC samples. Kaplan–Meier method was used to calculate survival curves, and the significance was analyzed by log-rank test. *P* < 0.05 was considered significant.

## Results

### Expression and circRNA characterization of circ_0000140 in OSCC and its association with patient prognosis

**Fig. 1:**
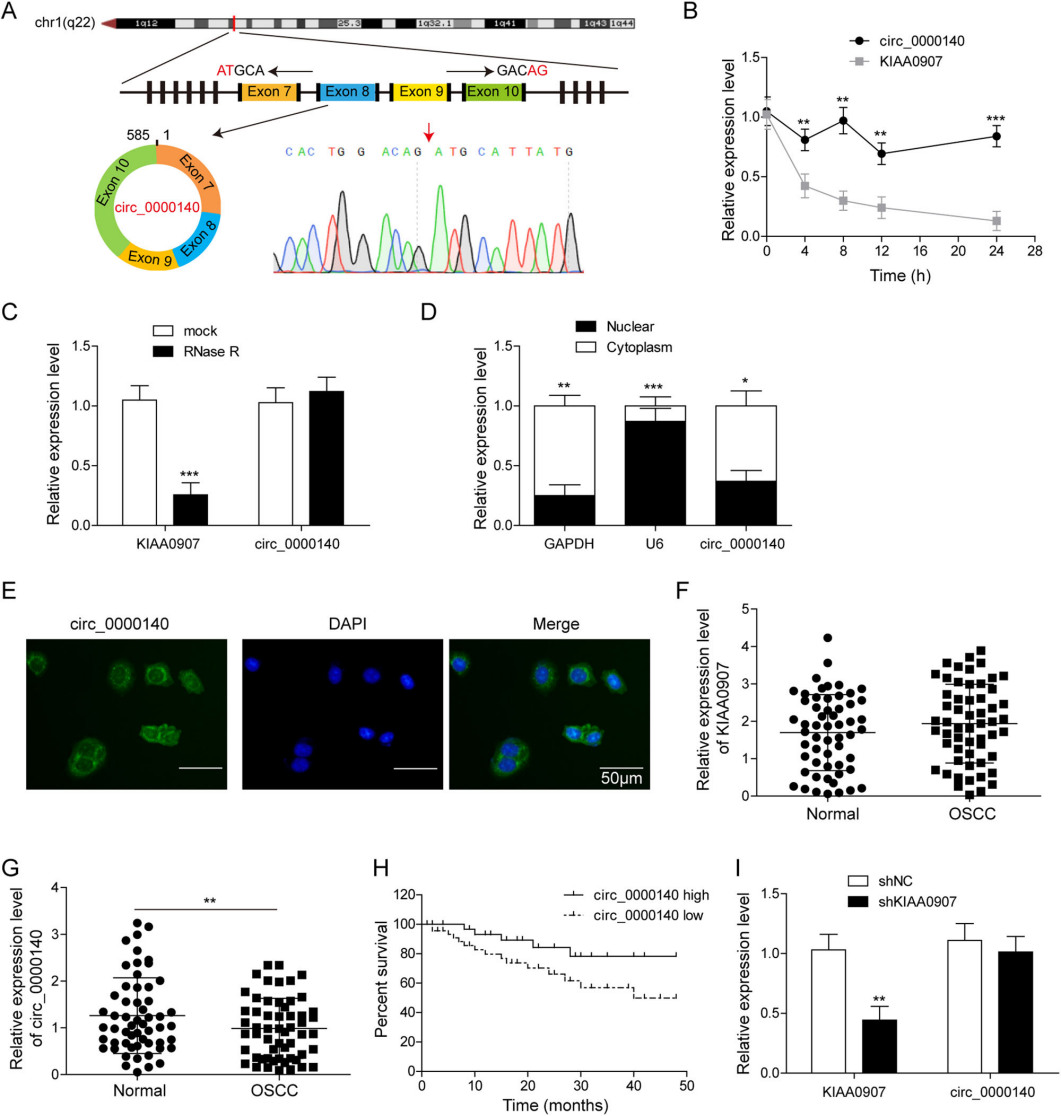
Expression and circRNA characterization of circ_0000140 in OSCC. **a** The exonic information of circ_0000140 was illustrated as indicated. The specific primers of circ_0000140 were validated by Sanger sequencing. The length of circ_0000140 was 585 bp. The red arrow indicates the backsplice site. **b** The relative RNA levels were examined by qRT-PCR after treatment with actinomycin D at the indicated time points in HOK cells. **c** The relative RNA levels were examined by qRT-PCR after treatment with RNase R or mock in total RNAs derived from HOK cells. **d** The cellular distribution of circ_0000140 was analyzed by cellular RNA fractionation assays. GAPDH and U6 were used as cytoplasmic and nuclear positive controls, respectively. **e** The cellular distribution of circ_0000140 was analyzed by fluorescence in situ hybridization (FISH). Green indicates circ_0000140. Nuclei were stained with DAPI. Scale bar, 50 μm. The levels of KIAA0907 (**f**) and circ_0000140 (**g**) in 56 paired OSCC and matched adjacent normal tissues were examined by qRT-PCR. **h** Kaplan–Meier method with the log-rank test was used to analyze the overall survival of OSCC patients in high and low circ_0000140 expression groups. **i** The relative expression levels were examined by qRT-PCR after treatment with shKIAA0907. All the results were shown as mean ± SD. *P < 0.05, **p < 0.01, and ***p < 0.001.

circ_0000140 is derived from exons 7 to 10 of the *KIAA0907* gene, whose spliced mature sequence length is 585 bp. The result of Sanger sequencing confirmed the head-to-tail splicing in the qRT-PCR product of circ_0000140 (Fig. 1a). Next, we investigated the stability and localization of circ_0000140 in HOK cells. Total RNAs from HOK cells were isolated after treatment with the transcription inhibitor actinomycin D. Then, qRT-PCR was performed to measure the level of circ_0000140 and KIAA0907 mRNA. The results showed that the half-life of circ_0000140 exceeded 24 h, whereas that of KIAA0907 mRNA was about 4 h in HOK cells, demonstrating that circ_0000140 was more stable than KIAA0907 (Fig. 1b). Furthermore, we found that compared with KIAA0907, circ_0000140 was significantly resistant to RNase R, implying that circ_0000140 was a circRNA (Fig. 1c). In addition, we found that circ_0000140 was predominately distributed in the cytoplasm of OSCC cells through cellular RNA fractionation (Fig. 1d) and fluorescence in situ hybridization (Fig. 1e).

To investigate clinical relevance of circ_0000140 in OSCC, we first analyzed the relationship between circ_0000140 expression and clinical features in 56 patients with OSCC. We found that low circ_0000140 levels were significantly correlated with higher lymph node metastasis (*P* = 0.015) and more advanced TNM (tumor, node, metastasis) stage (*P* = 0.031) in OSCC patients. On the other hand, circ_0000140 expression level was not associated with other parameters, including gender (*P* = 0.781) and age (*P* = 0.403) in OSCC (Table 1). We then analyzed the expression levels of KIAA0907, circ_0000140, miR-31, LATS1, and LATS2 in 56 pairs of OSCC tissues and matched adjacent normal tissues by qRT-PCR. As shown in Fig. 1f, no significant difference was found between OSCC tissues and matched normal tissues for the mRNA levels of KIAA0907. By contrast, the expression level of circ_0000140, LATS1, and LATS2 was significantly down-regulated and miR-31 expression was markedly increased in OSCC tissues (*P* < 0.001; Fig. 1g and Fig. S1A–C). Moreover, Kaplan–Meier and log-rank test analyses demonstrated that lower circ_0000140 expressions were associated with poor overall survival (*P* < 0.001; Fig. 1h). While knockdown of KIAA0907 dramatically reduced the expression level of KIAA0907 in HOK cells, it had no effect on the expression level of circ_0000140 (Fig. 1i). Linear regression analyses showed that circ_0000140 expression negatively correlated with miR-31 expression (*r*^2^ = 0.43, *P* < 0.001; Fig. S1D) and positively correlated with LATS2 expression (*r*^2^ = 0.60, *P* < 0.001; Fig. S1E), and that miR-31 expression negatively correlated with LATS2 expression (*r*^2^ = 0.54, *P* < 0.001; Fig. S1F). Western blotting analysis revealed the decreased protein levels of p53, LATS1, LATS2, YAP1, and phosphorylated form of YAP1 (p-YAP1) in OSCC tumor tissues (Fig. S1G, H). We then performed qRT-PCR analysis to detect the expression of KIAA0907, circ_0000140, and miR-31 in four OSCC cell lines (Cal-27, SCC-25, SCC-9, and HSC-3) and HOK cells (Fig. S1I–K). As expected, KIAA0907 expression did not differ between OSCC and HOK cells (Fig. S1I). circ_0000140 exhibited significantly low levels, whereas miR-31 expressed significantly high levels in all OSCC cell lines compared to HOK cells (Fig. S1J, K). As Cal-27 and HSC-3 cells showed the lowest expression of circ_0000140 and highest expression of miR-31, these two cell lines were selected for following studies. Overall, these results suggested potential involvement of circ_0000140, LATS2, and miR-31 in OSCC.

### circ_0000140 directly targets miR-31 in OSCC cells

**Fig. 2:**
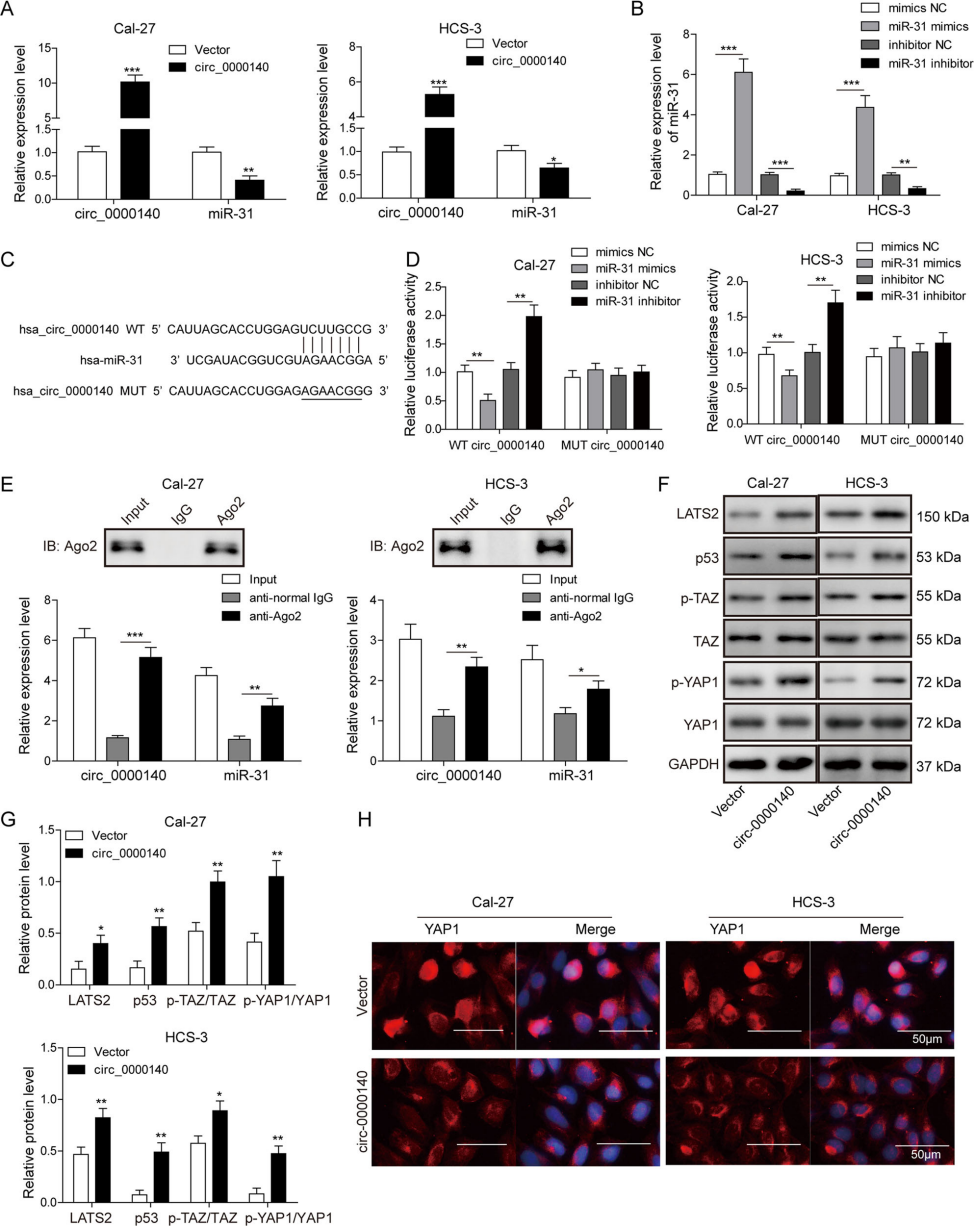
circ_0000140 directly targeted miR-31 in OSCC cell lines. **a** The expression level of circ_0000140 and miR-31 in Cal-27 and HSC-3 cell lines transfected with circ_0000140 overexpression vector. **b** The expression level of miR-31 in Cal-27 and HSC-3 cell lines transfected with miR-31 mimics, miR-31 inhibitors, and negative control. **c** The targeting sequence of circ_0000140 and miR-31. **d** Dual luciferase assay of miR-31 mimics, miR-31 inhibitors, and negative control on circ_0000140 wild type and mutated type. **e** Anti-AGO2 RIP assays were used in Cal-27 and HSC-3 cells to determine circ_0000140 and miR-31 RNA enrichment in IP complexes. Anti-IgG was used as a control. **f**, **g** Protein levels of the indicated Hippo pathway in circ_0000140-overexpressing OSCC cells, as detected by Western blotting. **h** Expression levels of YAP1 in circ_0000140-overexpressing OSCC cells as detected by immunofluorescence analyses. Scale bar, 50 μm. All the results were shown as mean ± SD (*n* = 3), which were three separate experiments performed in triplicate. *P < 0.05, **p < 0.01, and ***p < 0.001.

We next up-regulated the expression of circ_0000140 in Cal-27 and HSC-3 cells. Results of qRT-PCR analysis showed that circ_0000140 expression was significantly up-regulated by 10- and 5-fold, while miR-31 expression was significantly decreased by 60% and 40% in circ_0000140-overexpressing Cal-27 and HSC-3 cells, respectively, as compared with control cells (Fig. 2a). Furthermore, qRT-PCR analysis indicated that miR-31 overexpression led to a significant increase in miR-31 level, while depletion of miR-31 yielded opposite effects in both Cal-24 and HSC-3 cells (Fig. 2b). Bioinformatics analysis revealed that circ_0000140 contained a putative binding site of miR-31 (Fig. 2c). We then performed dual luciferase reporting assay to identify the interaction between miR-31 and the predicted circ_0000140 targeting sequences. As shown in Fig. 2d, the miR-31 mimics reduced the luciferase activity in the wild type rather than mutated type of circ_0000140 in both Cal-27 and HSC-3 cell lines, and the miR-31 inhibitors showed opposite effect in these cell lines. RIP assay was further performed using anti-AGO2 in the Cal-27 and HSC-3 cells, and it was found that circ_0000140 and miR-31 were enriched preferentially in miRNPs containing AGO2 compared with anti-IgG immunoprecipitates (Fig. 2e). Western blotting analysis showed that enhanced circ_0000140 led to an increase in LATS2, p53, p-TAZ, and p-YAP1 protein levels, while it had no effect on TAZ and YAP1 protein levels (Fig. 2f–g). Immunofluorescence analysis demonstrated that nuclear translocation of YAP1 protein was attenuated in circ_0000140-overexpressing OSCC cells (Fig. 2h), suggesting the potential suppressive effect of circ_0000140 on the Hippo signaling pathway. Taken together, these data demonstrated that circ_0000140 could directly target miR-31 in OSCC cells.

### circ_0000140 regulates proliferation, migration, invasion, and EMT process by targeting miR-31 in OSCC cells

**Fig. 3:**
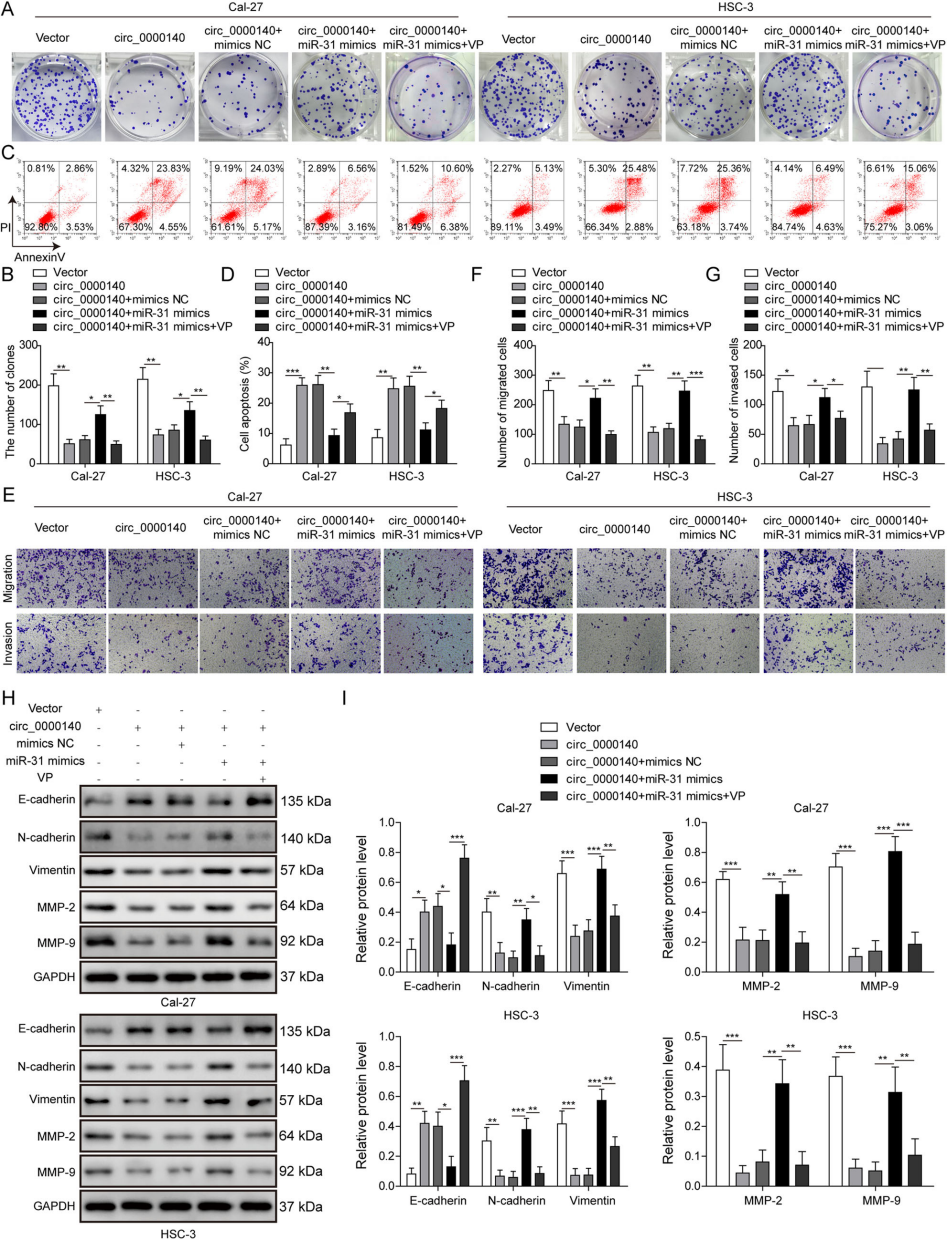
circ_0000140 regulated OSCC cancer proliferation, migration, invasion, and EMT process by directly targeting miR-31. **a**, **b** Proliferation of Cal-27 and HSC-3 cells transfected with circ_0000140 overexpression vector, miR-31 mimics, or verteporfin treatment as detected by colony formation assay. **c**, **d** Apoptosis of Cal-27 and HSC-3 cells transfected with different treatments, as detected by Annexin V/PI assay. **e–g** Migration and invasion of Cal-27 and HSC-3 cells transfected with different treatments, as detected by transwell assay. **h**, **i** Protein levels of the indicated EMT markers in Cal-27 and HSC-3 cells transfected with different treatments, as detected by Western blotting. All the results were shown as mean ± SD (*n* = 3), which were three separate experiments performed in triplicate. *P < 0.05, **p < 0.01, and ***p < 0.001.

**Fig. 4:**
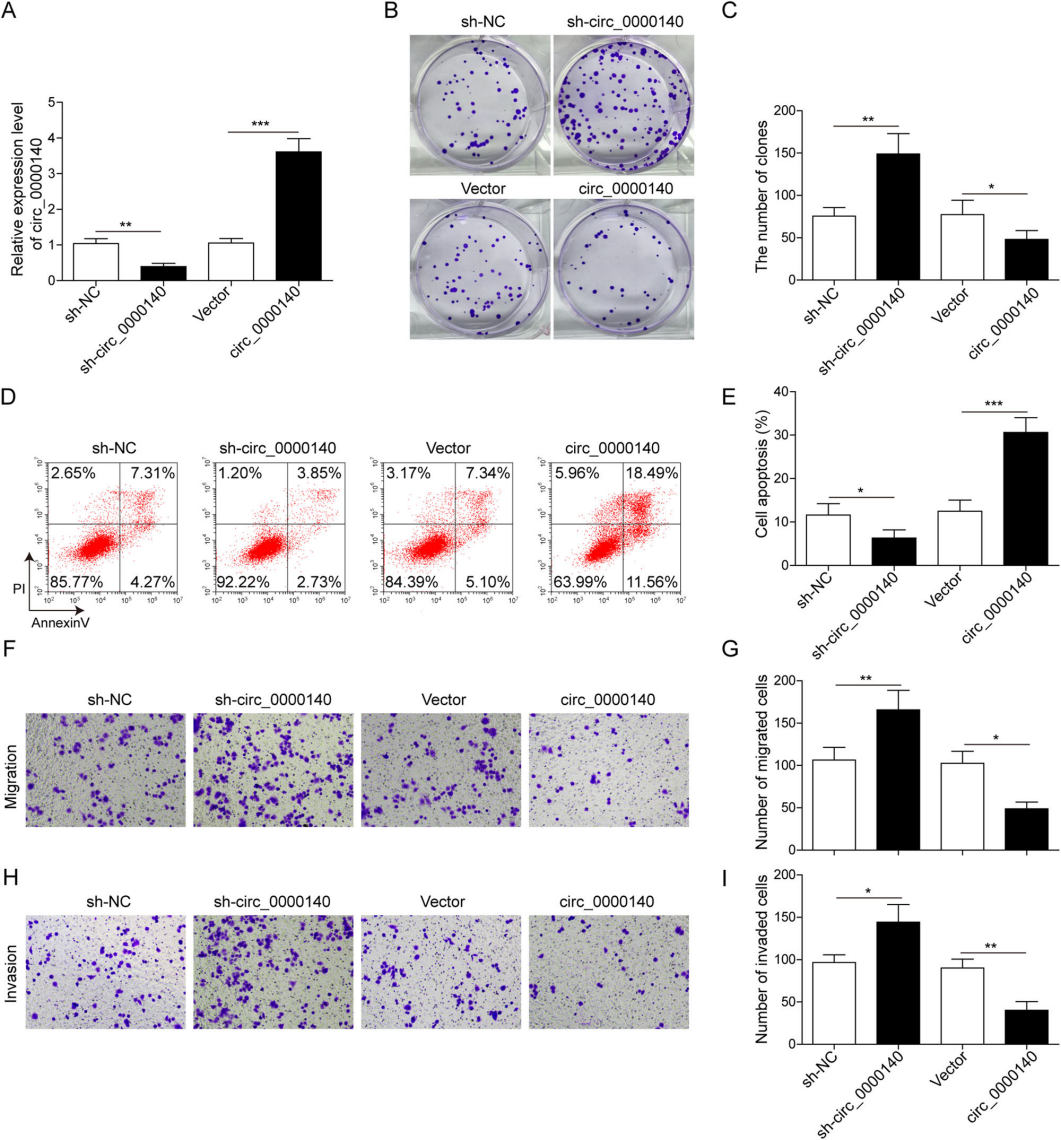
circ_0000140 inhibited proliferation, migration, invasion, and promoted apoptosis in HOK cells. **a** The expresssion levels of circ_0000140 in HOK cells transfected with sh-circ_0000140 or circ_0000140 overexpression vector as detected by qRT-PCR. **b**, **c** Proliferation of HOK cells transfected with sh-circ_0000140 or circ_0000140 overexpression vector as detected by colony formation assay. **d**, **e** Apoptosis of HOK cells transfected with sh-circ_0000140 or circ_0000140 overexpression vector as detected by Annexin V/PI assay. **f**, **g** Migration of HOK cells transfected with sh-circ_0000140 or circ_0000140 overexpression vector as detected by transwell assay. **h**, **i** Invasion of HOK cells transfected with sh-circ_0000140 or circ_0000140 overexpression vector as detected by transwell assay. All the results were shown as mean ± SD (*n* = 3), which were three separate experiments performed in triplicate. *P < 0.05, **p < 0.01, and ***p < 0.001.

To assess the effect of circ_0000140 on OSCC cell phenotype, we performed gain-of-function investigations by transfecting the vector that contained circ_0000140 overexpression in OSCC cells. Results from colony formation assay showed that Cal-27 and HSC-3 cells transfected with circ_0000140 overexpression vector displayed markedly lower colony-forming activity than the empty vector (Fig. 3a, b). As expected, circ_0000140 overexpression significantly enhanced cell apoptosis in both Cal-27 and HSC-3 cells (Fig. 3c, d). Moreover, cell migratory and invasive ability were reduced remarkably in circ_0000140-overexpressing cells, as measured by transwell assays (Fig. 3e–g). In order to functionally confirm that circ_0000140 suppresses progression of OSCC by sponging miR-31, we carried out rescue experiments by co-incubation of circ_0000140 overexpression vector with miR-31 mimics in OSCC cells. We found that overexpression of miR-31 effectively reversed the inhibitory effects of circ_0000140 overexpression on cell proliferation, migration, and invasion in vitro (Fig. 3a–g). Additionally, we first clarified the expression pattern and biological function of circ_0000140 in human normal oral keratinocyte cells by loss-of-function and gain-of-function experiments. The success of circ_0000140 knockdown through transfection of shRNA and circ_0000140 overexpression through transfection of circ_0000140 overexpression vector was confirmed by qRT-PCR analysis (Fig. 4a). As expected, results from colony formation, transwell assay, and Annexin V/PI flow cytometry showed that overexpression of circ_0000140 significantly inhibited the proliferation, migration, and invasion, and promoted apoptosis of HOK cells, whereas circ_0000140 silence exerted opposite effects (Fig. 4b–i). Moreover, while overexpression of circ_0000140 increased the level of E-cadherin and reduced the expression level of N-cadherin, vimentin, MMP-2, and MMP-9 in OSCC cells, overexpression of miR-31 antagonist suppressed the effect of circ_0000140 on EMT (Fig. 3h, i). Importantly, we found that restoration of a series of cell activities, as well as the EMT process by co-expressing miR-31 mimics in Cal-27 and HSC-3 cells expressing circ_0000140, was canceled by the treatment of verteporfin (VP), a YAP inhibitor (Fig. 3a–i). Overall, these results indicated that circ_0000140 suppressed cell proliferation, migration, invasion, and EMT of OSCC cells by targeting miR-31 in vitro.

### miR-31 directly targets LATS2 to activate Hippo signaling pathway in OSCC cells

**Fig. 5:**
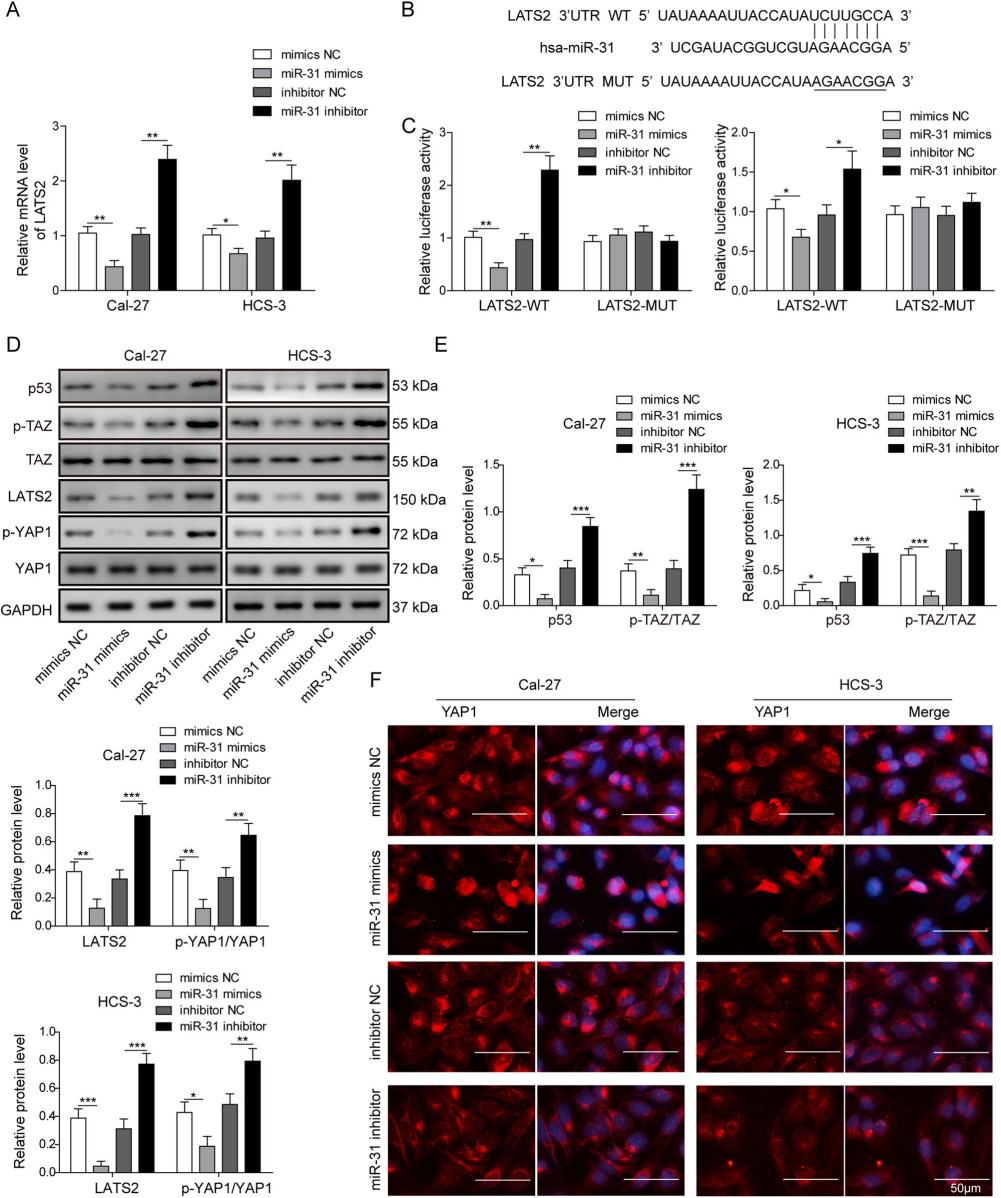
miR-31 activated Hippo pathway by directly targeting LATS2 in OSCC cell lines. **a** The effect of miR-31 mimics and inhibitor on LATS2 mRNA level in Cal-27 and HSC-3 cells, as determined by qRT-PCR. **b** The targeting sequence of LATS2 mRNA and miR-31. **c** Dual luciferase assay of miR-31 mimics, miR-31 inhibitors, and negative control on LATS2 wild type and mutated type. **d**, **e** The effect of miR-31 on p53 and Hippo pathway-related proteins by Western blotting. **f** Expression levels of YAP1 in miR-31-overexpressing or miR-31-underexpressing OSCC cells as detected by immunofluorescence analysis. Scale bar, 50 μm. All the results were shown as mean ± SD (*n* = 3), which were three separate experiments performed in triplicate. *P < 0.05, **p < 0.01 and ***p < 0.001.

To investigate the influence of miR-31 activity on LATS2 activation in OSCC cell lines, we performed gain-of-function and loss-of-function experiments by transfecting OSCC cells with miR-31 mimics or inhibitors. We found an apparent decrease in LATS2 expression in Cal-27 and HSC-3 cells transfected with the miR-31 mimics, whereas a marked increase in the level of LATS2 was found in both cells transfected with the miR-31 inhibitors (Fig. 5a), indicating that miR-31 negatively regulates LATS2 stability at the translational level. We then performed bioinformatics analysis to identify the potential of LATS2 as one target gene of miR-31 in OSCC cells. The results revealed that the LATS2-encoded mRNA contains a 3′-UTR element that is complementary to miR-31 (Fig. 5b). Dual luciferase reporter assay further showed that the miR-31 overexpression reduced 70% luciferase activity in cells transfected with wide-type LATS2 3′-UTR, while loss of miR-31 significantly enhanced the luciferase activities by as many as 2.5-folds in these cells. By contrast, no apparent effect was observed on the luciferase activity in cells transfected with mutant LATS2 3′-UTR (Fig. 5c). Since LATS2 activity is suppressed by miR-31, it is thus plausible that the phosphorylation levels of downstream signaling proteins was regulated by LATS2 decrease. As expected, Western blotting analysis showed that enhanced miR-31 led to a decrease in p53 and p-YAP1, but not YAP, while loss of miR-31 exerted an opposite effect (Fig. 5d, e). Notably, consistent changes were also observed in the protein level of phosphorylated TAZ, a well-known target of LATS kinases in the canonical Hippo pathway (Fig. 5d, e). Immunofluorescence analysis demonstrated the enhanced nuclear translocation of YAP1 protein in OSCC cells with miR-31 overexpression, while miR-31 knockdown led to an opposite effect (Fig. 5f), suggesting the potential promoting effect of miR-31 on the Hippo signaling pathway. Taken together, these results provided strong evidence for the direct targeting of LATS2 mRNA by miR-31, and miR-31 could activate Hippo signaling pathway in OSCC cells.

### Suppression of miR-31 inhibits cell proliferation, migration, and invasion in OSCC cells via directly down-regulating LATS2

**Fig. 6:**
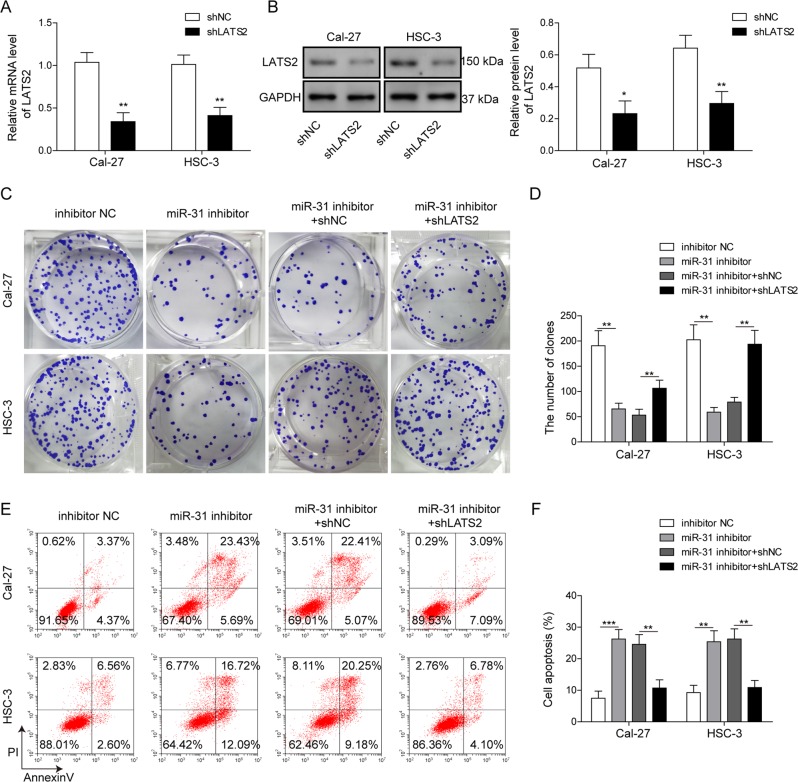
miR-31 accelerated proliferation and repressed apoptosis by directly targeting LATS2 in OSCC cells. **a**, **b** Knockdown of LATS2 by shRNA in Cal-27 and HSC-3 cells as confirmed by qRT-PCR and Western blotting, respectively. **c**, **d** Proliferation of Cal-27 and HSC-3 cells transfected with miR-31 inhibitors or shLATS2, as detected by colony formation assay. **e**, **f** Apoptosis of Cal-27 and HSC-3 cells transfected with different treatments, as detected by Annexin V/PI assay. All the results were shown as mean ± SD (*n* = 3), which were three separate experiments performed in triplicate. *P < 0.05, **p < 0.01, and ***p < 0.001.

**Fig. 7:**
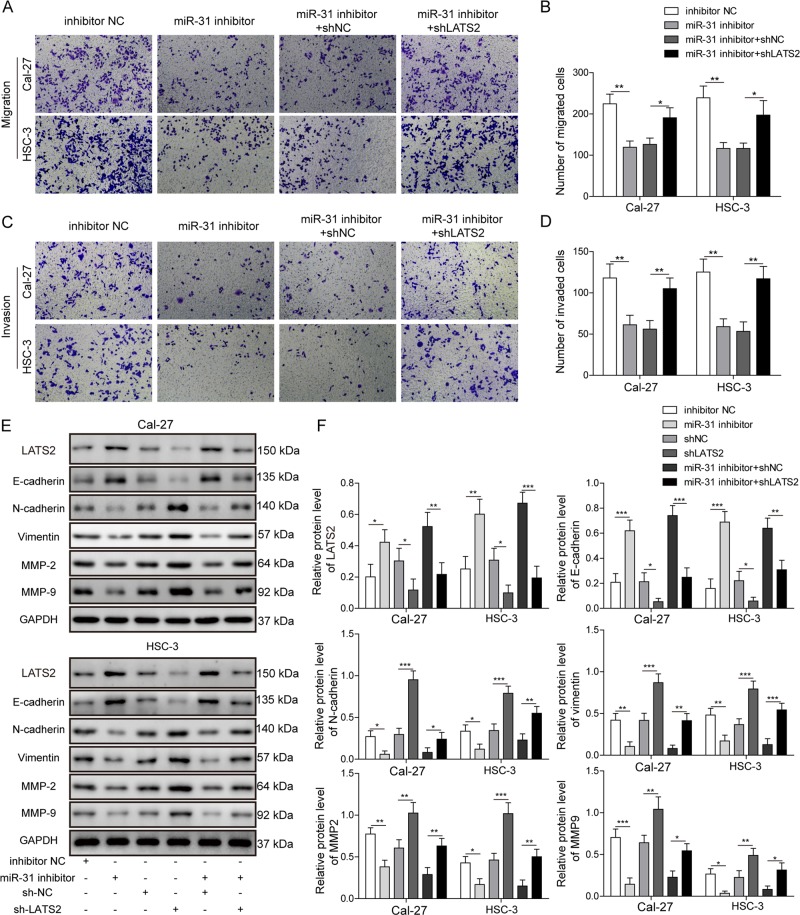
miR-31 promoted migration, invasion, and EMT by directly targeting LATS2 in OSCC cells. **a**, **b** Migration of Cal-27 and HSC-3 cells transfected with different treatments, as detected by transwell assay. **c**, **d** Invasion of Cal-27 and HSC-3 cells transfected with different treatments, as detected by the transwell assay. **e**, **f** Protein levels of the indicated LATS2 and EMT markers in Cal-27 and HSC-3 cells transfected with different treatments, as detected by Western blotting. All the results were shown as mean ± SD (*n* = 3), which were three separate experiments performed in triplicate. *P < 0.05, **p < 0.01, and ***p < 0.001.

To further determine the biological consequence of miR-31-regulated functions in OSCC, we conducted a series of loss-of-function experiments. qRT-PCR and Western blotting analysis confirmed the efficient shRNA-mediated knockdown of LATS2 (Fig. 6a, b). Depletion of miR-31 markedly reduced the cell proliferation and colony formation capacity (Fig. 6c, d). In vitro flow cytometry assay revealed that miR-31 depletion notably promoted the apoptosis of OSCC cells (Fig. 6e, f). Moreover, knockdown of LATS2 significantly reversed the effects of miR-31 inhibition on cell proliferation and apoptosis in OSCC cells (Fig. 6c–f). In addition, transwell migration assay demonstrated a strong correlation between miR-31 depletion and the decreased migratory and invasive potential of OSCC cells (Fig. 7a–d). We also found that miR-31 inhibition led to decreased protein levels of N-cadherin, vimentin, MMP-2, and MMP-9, and increased LATS2 and E-cadherin comparison with control cells (Fig. 7e, f). On the other hand, LATS2 knockdown alone inhibited E-cadherin expression while promoting the expression of N-cadherin, vimentin, MMP-2, and MMP-9 (Fig. 7e, f). Furthermore, knockdown of LATS2 attenuated the above changes induced by miR-31 inhibitors (Fig. 7e, f). Altogether, these data demonstrated that miR-31 acted positively on the progression of OSCC cells by inhibiting LATS2-mediated Hippo signaling pathway.

### circ_0000140 inhibits xenograft growth and lung metastasis in vivo

**Fig. 8:**
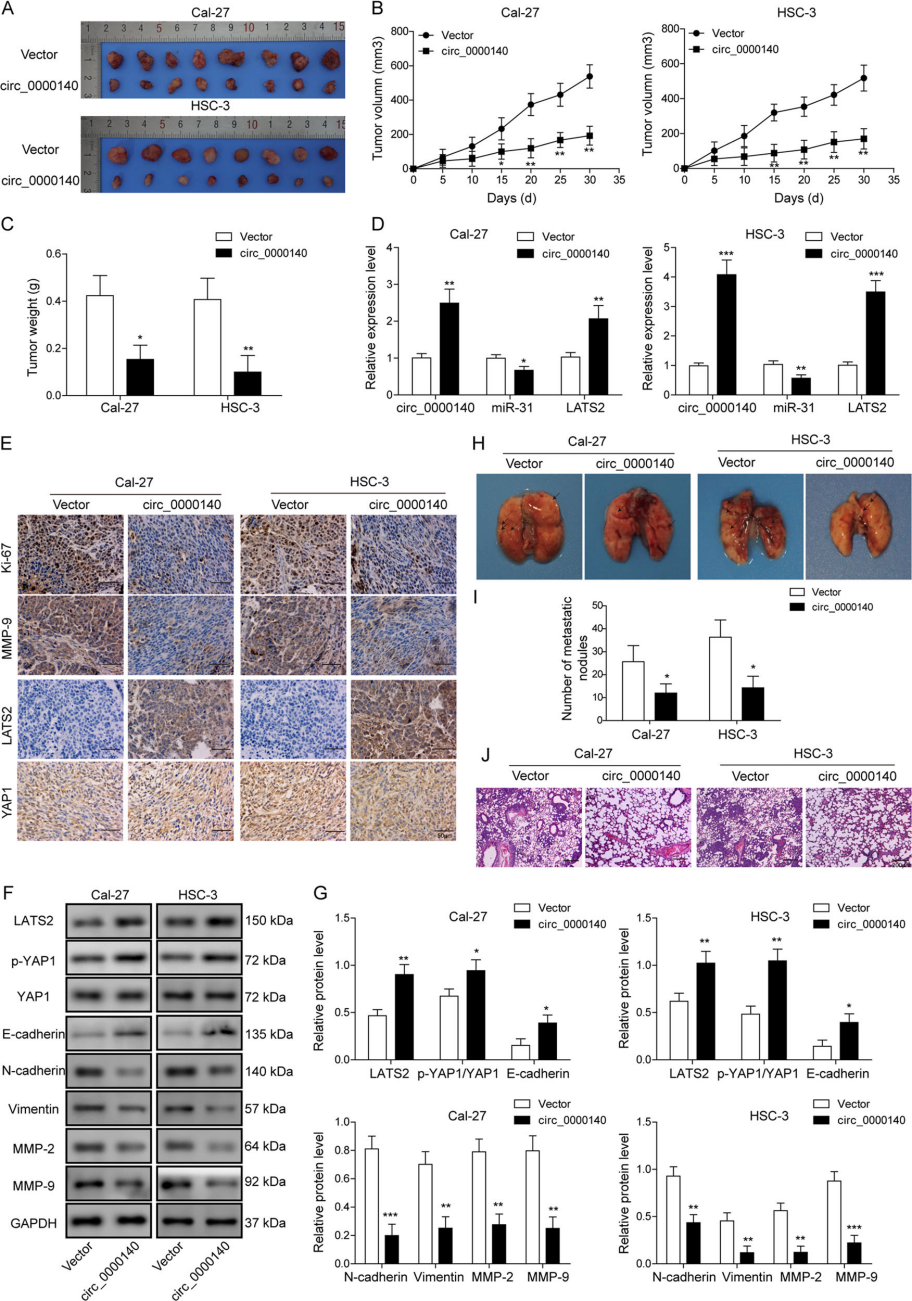
Overexpression of circ_0000140 inhibited the tumor growth and lung metastasis in nude mice. **a** Typical OSCC tumors from tumor-bearing BALB/c mice after subcutaneous injection of Cal-27 and HSC-3 cells stably overexpression circ_0000140. **b** The volume of OSCC tumors from tumor-bearing BALB/c mice after treatment. **c** The weight of OSCC tumors from tumor-bearing BALB/c mice after treatment. **d** The expression level of circ_0000140, miR-31, and LATS2 in xenograft tumor tissues as determined by qRT-PCR. **e** The protein level of Ki-67, MMP-9, LATS2, and YAP1 in xenograft tumor tissues as determined by immunohistochemistry. Scale bar, 50 μm. **f**, **g** The protein level of Hippo pathway and EMT markers in xenograft tumor tissues as determined by western blotting. **h** Typical lung tissues with visualized metastatic nodules from tumor-bearing BALB/c mice after tail vein injection of Cal-27 and HSC-3 cells stably overexpressing circ_0000140. **i** The number of metastatic nodules of lungs from tumor-bearing BALB/c mice after treatment. **j** H&E for metastatic nodules of lungs in circ_0000140 overexpression group and vehicle group. Scale bar, 200 μm. All the results were shown as mean ± SD. *P < 0.05, **p < 0.01, and ***p < 0.001.

Finally, to investigate whether circ_0000140 might regulate OSCC xenograft growth in vivo, Cal-27 and HSC-3 cells with stable overexpression of circ_0000140 were subcutaneously injected into athymic nude mice, respectively. Compared to those of control xenografts, circ_0000140 overexpression slowed down the tumor growth in nude mice by size and weight by more than 2-fold over a period of 30 days (Fig. 8a–c). qRT-PCR analysis confirmed the down-regulation of miR-31 and the up-regulation of LATS2 in circ_0000140-overexpressing cells (Fig. 8d). Immunohistochemistry results showed that LATS2 protein levels were higher in circ_0000140 overexpression group (Fig. 8e). Moreover, cytoplasmic retention of YAP1 protein was promoted by overexpressing circ_0000140 (Fig. 8e). Correspondingly, Ki-67 and MMP-9 protein levels were lower in cells with circ_0000140 enhancement (Fig. 8e).

To further analyze the expression level of LATS2 and its downstream protein p-YAP1, Western blotting was performed in the tumor tissues (Fig. 8f, g). As expected, both the protein levels of LATS2 and its down-regulating target p-YAP1 were enhanced in circ_0000140 overexpression group. Consistent with results from cell experiments, it was argued that circ_0000140 led to a dramatic increase in E-cadherin protein, as well as a reduction in N-cadherin, vimentin, MMP-2, and MMP-9 proteins in vivo (Fig. 8f, g). Most importantly, lungs in nude mice were harvested and metastasis was examined. The results showed that overexpression of circ_0000140 reduced the metastatic nodules of lungs by more than 2-fold (Fig. 8h, i). The hematoxylin and eosin staining of lung slides also suggested a marked reduction in the area of lung metastasis in the circ_0000140 overexpression group (Fig. 8j). Collectively, the nude mice experiments showed that circ_0000140 inhibited tumor formation and lung metastasis via EMT possessed by targeting miR-31 to repress LATS2-mediated Hippo signaling pathway.

## Discussion

The incidence of distant metastasis plays a critical role in the management and prognosis in patients with OSCC, with lung metastasis being the most frequent site that accounts for ~70% of cases^26^. A good understanding of the molecular mechanism underlying distant metastasis could thus advance the development of diagnostic and therapeutic strategies targeted to this usually terminal stage for OSCC patients. In the present study, we identified an OSCC-associated altered circRNA and further clarified the distinct cellular pathways regulated by this circRNA. Specifically, circ_0000140 directly targeted miR-31 and repressed the proliferative, migratory, and invasive capacity of OSCC cells. LATS2, one of the key components in the Hippo pathway and a direct target of miR-31, served as the key mediator of circ_0000140 function by suppressing EMT of OSCC. Importantly, clinical relevance of circ_0000140 was confirmed by the observed suppression in OSCC tumorigenic and lung metastasis in vivo, which is in accordance with cell experiments and also indicative of its value as a promising disease biomarker.

Owing to the structural stability, specificity, and accessibility of circRNAs, a growing number of reports have highlighted the potential of these non-coding RNAs as non-invasive biomarkers for cancer diagnosis and therapeutics^27^. Among them, several ones have been identified as potential biomarkers in OSCC, including the down-regulation of circ_0008309^17^ and circ_001242^28^, and the up-regulation of circ_0001874 and circ_0001971^19^. Although aberrant expression of circ_0000140 has been implicated in OSCC, its functions remain to be fully explored. To this end, our study in clinical samples demonstrated that circ_0000140 was down-regulated and the impaired circ_0000140 expression was significantly associated with advanced TMN stage and lymph node metastasis in patients with OSCC. Furthermore, survival analyses demonstrated a significantly shortened 5-year survival rate in OSCC patients with low circ_0000140 expression. In accordance with this finding, overexpression of circ_0000140 was able to inhibit cell proliferation, migration, and invasion in OSCC in vitro. We also revealed that circ_0000140 disturbed the EMT expression in OSCC, reflected by the increased E-cadherin and decreased N-cadherin protein in circ_0000140-overexpressing OSCC cells. In mouse model, overexpression of circ_0000140 not only reduced the tumor size but also inhibited the lung metastasis of tumor generated by two different OSCC cancer cell lines. In particular, the in vivo experiment of metastasis showed that circ_0000140 overexpression led to >50% reduction in metastatic nodules of lungs. These results suggested that dysregulation of circ_0000140 exerts a suppressive role in OSCC development.

As one of the most widely studied functions of circRNAs as miRNA sponges in the cytoplasm^29,30^, several circRNA-miRNA interplays have been shown to critically contribute to OSCC initiation and progression. For instance, hsa_circ_0008309 was firstly explored to suppress miR-136-5p and miR-382-5p expression and enhance ATXN1 expression in OSCC cells^17^. circDOCK1 was also enhanced in OSCC and suppressed cell apoptosis by functioning as ceRNA for the miR-196-5a^31^. circRNA_100290 was identified as a ceRNA to antagonize miR-378a-mediated GLUT1 inhibition, resulting in an induction of glycolysis and cell proliferation in OSCC^18^. In addition, circ-PKD2 weakened the tumor-promoting effects of miR-204-3p-driven APC2 on OSCC development through various signaling pathways^32^. Based on our bioinformatics data, we assumed that there was a binding site of miR-31 on circ_0000140. miR-31 is among the most frequently altered microRNAs in cancer and aberrant miR-31 expression has been detected in a series of tumor types^21^. Interestingly, miR-31 appears to be almost equally up- and down-regulated, depending on the cancer type. miR-31 has been found to be up-regulated in OSCC and to act as an oncogenic miRNA, for example, by targeting SIRT3 to disrupt mitochondrial activity or inhibiting ACOX1 to enhance cell motility^24,33^. Consistently, we found that miR-31 was significantly up-regulated in OSCC tissues and cells, and circ_0000140 could directly bind to miR-31 in OSCC cells. We then performed the gain-of-function experiments and established the regulatory relationship between circ_0000140 and miR-31. In particular, rescue experiments in vitro demonstrated that miR-31 could counteract the tumor-repressing effects, together with the suppression of EMT induced by overexpression of circ_0000140 in OSCC cells. Our study thus further provided an example of circRNA-miRNA network in oral cancer proliferation, migration, and invasion and EMT process.

LATS2 is a core kinase component of the Hippo tumor-suppressive signaling pathway that encodes a Ser/Thr protein kinase and LATS2-mediated YAP1 phosphorylation has been implicated in multiple human cancers^34^. For example, aberrant LATS2 overexpression has been observed and associated with aggressive features and unfavorable survival incolorectal cancer^35^, non-small-cell lung cancer^36^, and gallbladder cancer^37^. In the context of OSCC, LATS2 has been reported to inhibit cell proliferation and invasion by phosphorylating YAP, or act through promoter methylation^13,14^. While overexpression of miR-31 was shown to play an oncogenic role by repressing expression of LATS2, little is known about the role of miR-31/LATS2 signaling in regulating OSCC cells^38^. In our study, we found that LATS2 expression was markedly decreased in OSCC cells, and our luciferase reporter assay revealed that LATS2 could directly target miR-31. Moreover, knockdown of miR-31 could accelerate LATS2-mediated Hippo signaling pathway, as reflected by the increased level of YAP1 phosphorylation and the decreased nuclear translocation of YAP1 protein. In addition, shRNA-mediated LATS2 depletion efficiently reversed the repression of EMT induced by miR-31 inhibition. These results fully demonstrated that miR-31 directly targets the LATS2 to activate Hippo signaling pathway in oral cancer cells. Furthermore, we identified circ_0000140 as a novel regulator of the Hippo pathway in OSCC tumorigenesis and lung metastasis in vivo, as evidence by the induction of LATS2 and YAP1 phosphorylation. Consistent with the finding that miR-31 could modulate the LATS2/YAP1 expression, our results thus support that circ_0000140 suppresses OSCC cell growth and metastasis by regulating the Hippo pathway.

In conclusion, this is the first study demonstrating that circ_0000140 acts as a tumor suppressor that inhibits tumorigenesis and metastasis in OSCC, enforcing its inhibitory function by deactivating miR-31 to up-regulate the key component of the Hippo pathway network, LATS2. Our findings underscore that circ_0000140 has great potential to indicate the progression of OSCC, and that the Hippo signaling pathway may well be the candidate for intervention by molecular targeting therapeutics.

## Supplementary information

Fig. s1 legend

figure s1
