# Supplementary material for: circRNA_0000140 suppresses oral squamous cell carcinoma growth and metastasis by targeting miR-31 to inhibit Hippo signaling pathway
Source: Cell Death Dis. 2020 Feb 10;11(2):112. doi: 10.1038/s41419-020-2273-y (PMC7010827; doi:10.1038/s41419-020-2273-y)
Supplement: Supplementary file 1 — Fig. s1 legend [file 41419_2020_2273_MOESM1_ESM.docx]

**Figure s1. Expression level of** **circ_0000140, miR-31 and Hippo pathway-related proteins in OSCC tissues and cells.** The expression level of miR-31 (A), LATS2 (B), and LATS1 (C) in OSCC tumor tissues and normal tissue as determined by qRT-PCR (n=56). (D-F) Associations between circ_0000140, miR-31 and LATS2 expression in OSCC samples as assessed by spearman correlation analysis. (G-H) The protein level of p53 and Hippo pathway in OSCC tumor tissues and normal tissue as determined by Western blotting. The expression level of KIAA0907 (I), circ_0000140 (J) and miR-31 (K) in four OSCC cancer cell lines (Cal-27, SCC-25, SCC-9 and HSC-3) and human normal oral keratinocytes HOK as determined by qRT-PCR. All the results were shown as mean ± SD. * = p< 0.05 and ** = p < 0.01.
