# Supplementary figures and images for: circRNA_0000140 suppresses oral squamous cell carcinoma growth and metastasis by targeting miR-31 to inhibit Hippo signaling pathway
Source: Cell Death Dis. 2020 Feb 10;11(2):112. doi: 10.1038/s41419-020-2273-y (PMC7010827; doi:10.1038/s41419-020-2273-y)

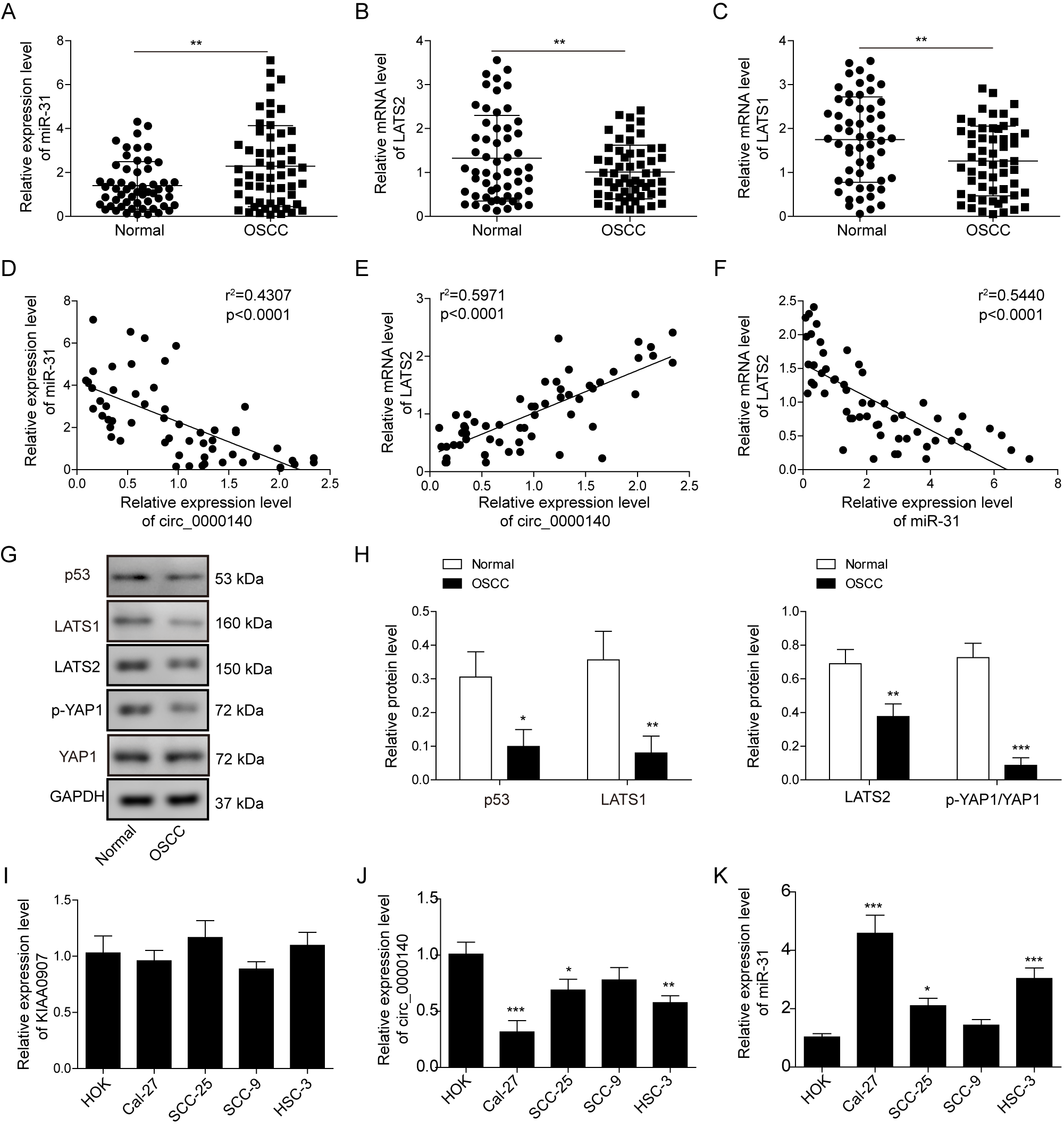

Supplement: Supplementary file 2 — figure s1 [file 41419_2020_2273_MOESM2_ESM.tif]
